# Simultaneous Assessment of Urinary and Fecal Volatile Organic Compound Analysis in De Novo Pediatric IBD

**DOI:** 10.3390/s19204496

**Published:** 2019-10-16

**Authors:** Sofia el Manouni el Hassani, Sofie Bosch, Jesse P.M. Lemmen, Marina Brizzio Brentar, Ibrahim Ayada, Alfian N. Wicaksono, James A. Covington, Marc A. Benninga, Nanne K.H. de Boer, Tim G.J. de Meij

**Affiliations:** 1 Department of Pediatric Gastroenterology, Emma Children’s Hospital, Amsterdam UMC, Academic Medical Center, 1081 HV Amsterdam, The Netherlands; m.a.benninga@amsterdamumc.nl; 2 Department of Pediatric Gastroenterology, Emma Children’s Hospital, Amsterdam UMC, Vrije Universiteit Amsterdam, 1105 AZ Amsterdam, The Netherlandsm.brizziobrentar@amsterdamumc.nl (M.B.B.);; 3 Department of Gastroenterology and Hepatology, Amsterdam Gastroenterology and Metabolism Research Institute, Amsterdam UMC, Vrije Universiteit Amsterdam, 1081 HV Amsterdam, The Netherlands; s.bosch1@amsterdamumc.nl (S.B.);; 4 School of Engineering, University of Warwick, Coventry CV4 7AL, UK; A.Wicaksono@warwick.ac.uk (A.N.W.); J.A.Covington@warwick.ac.uk (J.A.C.)

**Keywords:** biomarkers, inflammatory bowel disease, gas chromatography-ion mobility spectrometry

## Abstract

Endoscopic evaluation is mandatory in establishing the diagnosis of pediatric inflammatory bowel disease (IBD), but unfortunately carries a high burden on patients. Volatile organic compounds (VOC) have been proposed as alternative, noninvasive diagnostic biomarkers for IBD. The current study aimed to assess and compare the potential of fecal and urinary VOC as diagnostic biomarkers for pediatric IBD in an intention-to-diagnose cohort. In this cohort study, patients aged 4–17 years, referred to the outpatient clinic of a tertiary referral center under suspicion of IBD, were eligible to participate. The diagnosis was established by endoscopic and histopathologic assessment, participants who did not meet the criteria of IBD were allocated to the control group. Participants were instructed to concurrently collect a fecal and urinary sample prior to bowel lavage. Samples were analyzed by means of gas chromatography–ion mobility spectrometry. In total, five ulcerative colitis patients, five Crohn’s disease patients, and ten age and gender matched controls were included. A significant difference was demonstrated for both fecal (*p*-value, area under the curve; 0.038, 0.73) and urinary (0.028, 0.78) VOC profiles between IBD and controls. Analysis of both fecal and urinary VOC behold equal potential as noninvasive biomarkers for pediatric IBD diagnosis.

## 1. Introduction

Inflammatory bowel disease (IBD), classically divided into the two phenotypes—Crohn’s disease (CD) and ulcerative colitis (UC)—presents in childhood in approximately 25% of de novo cases and its incidence has increased over the past decades to ten per 100,000 children [1,2]. Establishing IBD diagnosis in this population can be challenging, since presenting clinical symptoms are commonly heterogeneous and nonspecific. To date, the gold standard for the diagnosis of IBD remains endoscopic evaluation and histologic examination of mucosal biopsies, however, this is an invasive procedure which carries a high burden on patients [3]. Fecal calprotectin (FCP) has been demonstrated as a useful noninvasive biomarker in the selection of patients eligible for further endoscopic evaluation, however, its specificity (68%) is limited, resulting in false-positive results and, subsequently, the performance of unnecessary colonoscopies [4]. Therefore, the search for noninvasive diagnostic IBD tests remains warranted [5].

Volatile organic compounds (VOC) have emerged as a promising alternative. These gaseous molecules are products of physiologic and pathophysiologic metabolism and are emitted from various bodily excrements. Alterations in cellular metabolic processes (e.g., in diseased state, microbiome metabolism, and microbiota-host interaction) are reflected by changes in the emitted VOC composition [6]. For VOC analyses, gas-chromatography-mass spectrometry (GC–MS) is considered the gold standard, because of its ability to identify specific VOC based on their physiochemical properties [7]. A relatively new method has been introduced for VOC analyses, being gas chromatography–ion mobility spectrometry (GC–IMS), in which traditional GC is combined with an orthogonal separation of molecules based on ion mobility [8]. By combining these two methods, the sensitivity for VOC analyses can be increased. 

Fecal VOC are most extensively studied in microbiome-associated gastro-intestinal diseases, since fecal VOC are largely produced during bacterial fermentation in the gut and reflect microbial composition, function, and microbiota–host interaction [9]. Fecal VOC analyses have demonstrated to allow for differentiation of a variety of diseases, including colorectal carcinoma, celiac disease, and IBD [5,10,11]. Similarly, testing of urinary VOC has potential to identify various diseases, such as cancer, diabetes and adult IBD [12,13,14,15]. IBD-related VOC can be detected in both exhaled breath and urine, however, exhaled breath analysis has shown to be challenging because of sample instability and the need for comprehensive analytical methods [7,16,17,18,19]. Collection of feces has been described to evoke feelings of embarrassment and concerns about hygiene by patients [20]. In most countries, however, collection of urine samples is considered as more user friendly and, therefore, less of a burden for patients [21]. Furthermore, urine can be more easily provided on demand in situations where fast analysis is required to accelerate the diagnostic process. Therefore, it is aimed in the current study to assess and compare the diagnostic accuracy of fecal and urinary VOC in de novo pediatric IBD by means of GC–IMS. 

## 2. Materials and Methods

### 2.1. Subjects

The current case-control pilot study was part of an ongoing cohort study, in which patients (aged 4–17 years) are included at the pediatric gastroenterology department outpatient clinic of a tertiary referral center (Amsterdam University Medical Centre; locations: VU medical center (VUmc) and Amsterdam medical center (AMC)) [22,23,24,25]. For the current study, patients suspected of IBD in the period May 2017 to February 2019 were eligible to participate. Exclusion criteria were a proven bacterial or viral gastroenteritis during the month prior to inclusion, use of immunosuppressive therapy, antibiotic or probiotic treatment in the last three months prior to inclusion, diagnosis with an immunocompromising disease, and insufficient ability to understand the Dutch language. Furthermore, participants who were not able to deliver both samples (i.e., feces and urine) were excluded.

Participants, and in case of participants aged under 12 years, parents, were asked for informed consent after their first visit to the outpatient ward. The Medical Ethical Review Committee of the VUmc approved the study protocol (file number 2015.393, amendment number A2017.188).

All patients underwent diagnostic endoscopic evaluation because of suspected IBD based on clinical symptoms and/or biochemical abnormalities (e.g., elevated FCP). Participants were allocated to either the IBD or control group, based on the combination of endoscopy, histology, biochemical, and radiological findings, according to the currently applied international diagnostic criteria [1]. Disease localization and behavior in IBD cases were assessed based on the Paris classification [26]. IBD cases were matched to controls based on age and gender. 

### 2.2. Sample and Data Collection

Participants were asked to concurrently collect a fecal and urine sample in containers (Stuhlgefäβ 10 mL, Frickenhausen, Germany), prior to bowel lavage. Participants were asked to store the samples in their freezer at home within one hour after collection and bring the samples to the hospital in cooled condition on their next regular appointment at the outpatient clinic. Samples were then directly stored at −20 °C until further handling. In addition to the sample collection, participants were asked to complete an online accessible and secured questionnaire (Castor EDC^®^) on dietary preferences, clinical symptoms, Bristol stool scale, bowel habits, extra-intestinal symptoms, residency, medical history, and medication use. Additional clinical information was collected from medical files, including IBD disease activity based on the physician global assessment (PGA) and laboratory values (CRP and FCP) [27,28]. 

### 2.3. Sample Preparation

Sample preparation was performed according to our standard operating procedure, previously described by Rouvroye et al. [29]. In short, a calibrated scale (Mettler Toledo, AT 261 Delta Range, Columbus, OH, USA) was used to weigh approximately 500 mg of fecal and frozen urine sample. Subsequently, the sample was transferred to a glass vial (20 mL, Frickenhausen, Germany), which was restored in a −20 °C freezer [22]. The fecal and urine samples were sent to the BioMedical Sensors Lab, School of Engineering, University of Warwick (Coventry, UK) on dry ice (−80 °C) for VOC analyses. 

### 2.4. Sample Analyses

Samples were analyzed following the methods by Rouvroye et al. [29]. Samples were randomly analyzed by means of GC-IMS (FlavourSpec^®^, G.A.S., Dortmund, Germany). This device consists of a GC column front-end, coupled with a drift tube IMS. The complex chemical mixture deriving from the sample headspace were pre-separated by the GC, before detection by IMS. Within the IMS, VOC ions were created by soft chemical-ionization (low-radiation tritium (H3) source). These ions were fed into a drift tube, which were propelled along it by an electric field. Against the flow of ions a buffer gas was added (in our case pure nitrogen). In general, larger molecules were struck more times than smaller molecules, losing momentum and, thus, taking longer to travel along the tube. As a result each substance’s drift time is dependent on the interaction of the ion with the electric field and the buffer gas. A Faraday plate was used to measure the resulting ion current, as a function of time [30]. The GC–IMS was connected to an automatic sampling system with a chiller allowing processing of a batch of 32 samples kept in cooled condition (4 °C) until the start of the analyses to minimize sample degradation (PAL RSi, CTC Analytics AG, Zwingen, Switzerland). In the eight minutes before analysis, the samples’ temperature was raised to 80 °C. Then, a syringe transported the headspace from the vial into the injector port of the instrument and into the GC column. Nitrogen 99.9% (3.5 bar), served as a carrier gas at 40 °C for GC separation, and as drift gas for IMS at 45 °C. GC flow rate was set at 20 mL/minute (34.175 kPa) for six minutes, while a 150 mL/min flow rate (0.364 kPa) was used for IMS. 

### 2.5. Statistical Analyses

The Statistical Package for the Social Sciences (SPSS, IBM version 22.0) was used to perform the statistical analyses on demographics. Patient demographics and clinical characteristics were compared using a Fisher’s exact test for dichotomous and ordinal data, and an independent t-test for parametric continuous data. In case of non-parametric continuous data, a Mann–Whitney U test was performed. A *p*-value < 0.05 was considered as a statistical significant difference. 

Prior to the statistical analyses of VOC profiles, the GC–IMS data was pre-processed to only crop areas containing relevant chemical data, which is located centrally within the data. Group noise was filtered after setting a threshold limit and, finally, a correction was performed for instrumental disturbances by baseline correction. This reduced the data points per sample from around 11 million to a more manageable 100,000. Classification was performed applying a 10-fold cross-validated approach, in which the data was split into a 90% training set and a 10% test set. The 100 most discriminatory features were identified by Wilcoxon rank-sum test (undertaken within the fold) and then used to train five different classifiers: random forest, Gaussian process, sparse logistic regression, support vector machine, and neural network algorithms. Subsequently, these models were applied to the test set. This process was repeated until every sample was classified as a test sample and from the resultant classification probabilities, statistical results were calculated. This method is part of our standard pipeline used in similar studies [18,29,31]. Statistical analyses were performed using R packages, including randomForest for random forest, glmnet for sparse logistic regression, kernlab for support vector machines, neuralnet for neural network analysis, and kernlab for Gaussian processes.

## 3. Results

### 3.1. Baseline Characteristics

**Figure 1:**
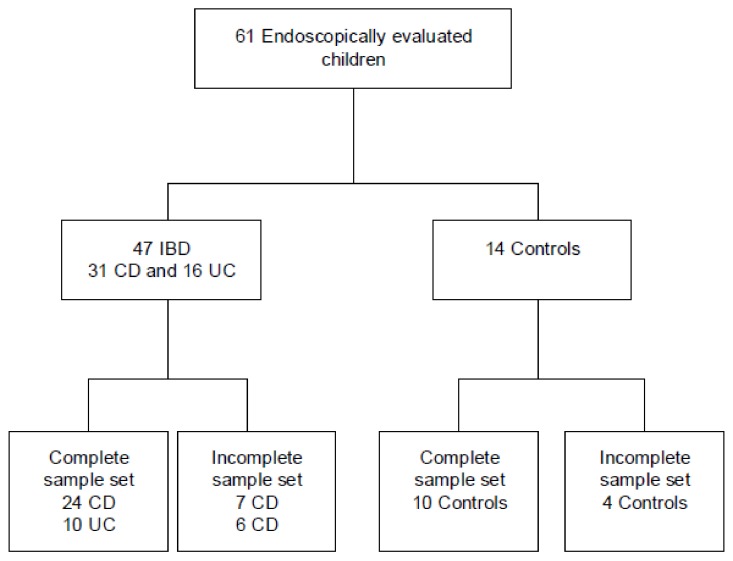
Inclusion flowchart Abbreviations: n, number; IBD, inflammatory bowel disease; CD, Crohn’s disease; UC, ulcerative colitis.

In total, ten IBD (five UC and five CD) and ten matched controls were included from the intention to diagnose cohort (Figure 1). The baseline characteristics are listed in Table 1. Fecal calprotectin was significantly higher (median 1208 µg/g, *p*-value 0.009) in the IBD group compared to the control group (median 50 µg/g). No differences were found for the remaining variables, as listed in Table 1.

In Table 2, the diagnoses established after diagnostic endoscopy in the control group are listed. Three children were diagnosed with irritable bowel syndrome or functional abdominal pain, three had an alternative diagnosis, and in four children no alternative diagnosis was established. Three children in the IBD group used prescribed medication, opposed to six children in the control group (Table 3).

### 3.2. VOC Analysis

**Figure 2:**
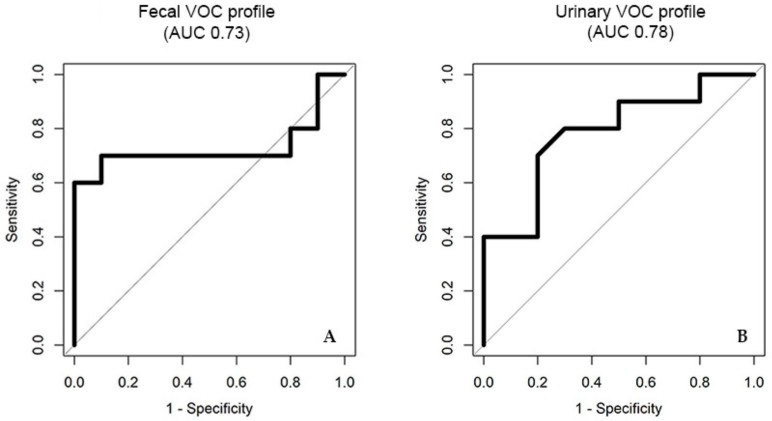
(**A**) Receiver operating characteristic (ROC) curves display the ROC curve for the fecal VOC profile, and (**B**) displays the ROC curve for urinary VOC profile for the discrimination of IBD from controls. Abbreviations: AUC, area under the curve.

**Figure 3:**
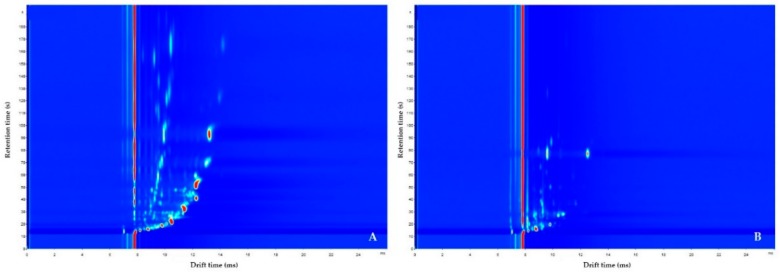
Examples of GC–IMS output. Here, the GC-IMS of fecal (**A**) and urinary (**B**) VOC profiles of one IBD patient are displayed. On the y-axis, the retention time indicates the separation time of the analyte by means of gas chromatography. The color represents the VOC concentration using color spectrum from blue to red, indicating low concentration to high concentration, respectively.

The discrimination of IBD from controls based on fecal VOC profiles, was statistically significant when training the algorithm using the neural network analysis (area under the curve (AUC) (95% confidence interval (CI)), p value, sensitivity, specificity; 0.73 (0.47–0.99), 0.038, 0.70, 0.90), Figure 2A. For urinary VOC profiles, a similar accuracy was reached for the discrimination between IBD and controls using the sparse logistic regression classifier (0.78 (0.57–1), 0.028, 0.80, 0.70), Figure 2B. The results of the remaining statistical analyses are listed in Supplementary Tables S1 and S2. An example of GC–IMS output of feces and urine, collected by the same participant, is displayed in Figure 3. 

## 4. Discussion

In the current study, the diagnostic accuracies of fecal and urinary VOC profiles to detect IBD were assessed and compared, using an endoscopy-controlled intention to diagnose cohort. Diagnostic accuracies were similar for both fecal and urine VOC profiles. 

The potential of fecal VOC analysis in pediatric IBD has been previously studied. Van Gaal et al. used field asymmetric ion mobility spectrometry (FAIMS) to analyze fecal VOC composition, which allowed for discrimination between 36 pediatric IBD patients and 24 healthy controls (HC) (AUC 0.76, sensitivity 0.79, specificity 0.79, *p*-value < 0.001) [23]. In another study on pediatric IBD, a robust difference in fecal VOC profiles between 26 UC patients and 28 HC (AUC, sensitivity, specificity, *p*-value; 1.00, 1.00, 1.00, <0.001) and between 29 CD and 28 controls (0.85, 0.86, 0.67, <0.001) were observed [25]. In the current study, similar diagnostic accuracies were identified for the discrimination of IBD from controls. However, due to smaller sample size, no sub-analyses could be performed to assess the diagnostic accuracy of fecal VOC analyses in the discrimination of both entities from controls. 

To the best of our knowledge, the current study is the first in which the potential of urinary VOC profiles in pediatric IBD is assessed. In adult IBD, urinary VOC analysis allowed for discrimination between IBD and HC by means of an eNose device (Fox 4000; AlphaMOS, Toulouse, France) and FAIMS [15]. The FAIMS analysis demonstrated similar diagnostic accuracies (AUC 0.75, *p*-value < 0.001), while VOC profiles analyzed by the eNose device (AUC 0.88, *p*-value < 0.001) exceeded the accuracy as observed in the current study. The variation in diagnostic accuracies can be explained by several aspects. In the current study, a pediatric intention to diagnose cohort was established, whereas in the study by Arasaradnam et al., adult patients with an established IBD diagnosis which received treatment were included. Additionally, IBD cases were compared to healthy controls, rather than with symptomatic patients, in which it is expected that there is a larger difference between healthy controls and cases, than is seen between symptomatic non IBD patients compared to IBD cases. Furthermore, different analytical techniques were applied, Arasaradnam et al. used an eNose device and FAIMS, whereas in the current study a novel technique, GC–IMS, was applied. Lastly, in the current study, a more diverse control group was included, which could possibly have led to a lower diagnostic accuracy. 

Since the algorithms applied in the current study have different internal learning structures and certain algorithms tend to perform better in different data collections, multiple algorithms were assessed in order to identify the best performing algorithm per bodily excrement [32]. Additionally, it is hypothesized that different VOCs can be detected in urine and feces. Analyses of fecal VOCs is believed to reflect the gut microbiota composition and local inflammatory processes. On the contrary, urinary VOCs associated with IBD are more likely to be a combination of compounds that diffused from the intestine into the bloodstream and VOCs that are associated with general inflammation, which therefore can be detected in urine [15,33]. However, to confirm this hypothesis, in-depth studies are needed to identify specific VOCs in both urine and feces. 

The main strength of this study is the inclusion of cases and controls from an endoscopy-controlled intention-to-diagnose cohort. Especially when aiming to find a novel non-invasive biomarker to prevent the performance of (unnecessary) endoscopic assessment of pediatric patients, it is important to include a realistic selection of patients with gastro-intestinal symptoms representing clinical practice, since this is the population that would potentially gain the most from an alternative diagnostic biomarker. In addition we included de novo IBD patients, circumventing bias by VOC altering effects of bowel lavage and immunosuppressive medication [34]. All controls were successfully matched to a case based on age and gender. Additionally, there were no differences in BMI between groups, which is a known factor affecting the VOC profile outcome [35]. By comparison of two different bodily excrements, concurrently collected by participants, we were able to identify the most suitable bodily excrement to analyze. Urine offers practical advantages compared to feces while maintaining a similar diagnostic accuracy and should, therefore, be considered as a clinically implementable bodily excrement for VOC analysis. To assess the diagnostic accuracy of fecal and urinary VOCs, we chose to perform the analysis by means of GC–IMS. These analyses are quick (approximately ten minutes per sample), sample pre-treatment is not required and the system setup is relatively easy [8,18,36]. Furthermore, machine learning algorithms can be trained to quickly recognize a disease specific profile without the need of the identification of individual VOC, making the GC–IMS output suitable for future clinical application. Lastly, there was no statistical difference between sample storage time between groups, which is demonstrated to affect VOC profile outcome [21,22].

This pilot study is limited by a relatively small sample size; consequently, no sub-analyses could be performed, assessing the diagnostic accuracy of fecal and urinary VOCs in the discrimination of both phenotypes from controls. Additionally, cases and controls could not be matched based on the possible confounding effect of medication use because of the variety of types of medication that were reported. One control patient was using esomeprazole, a proton pump inhibitor, that has been shown to influence the fecal VOC pattern [35,37]. Although the other reported medicines have not been described to affect the VOC profile, this difference might have influenced the VOC outcome.

Both fecal and urinary VOC analyses allowed for the discrimination of de novo IBD from controls by means of GC–IMS, with similar diagnostic accuracies, making them potential adjuvant biomarkers in the diagnostic work up of IBD. 

## Figures and Tables

**Table 1:** Baseline characteristics.

	IBD (n = 10)	Controls (n = 10)	*p*-Value
Sex male (n (%))	4 (40)	4 (40)	1.00
Age years (median (IQR))	15.0 (10.4–17.1)	14.2 (9.6–16.6)	0.71
BMI kg/m^2^ (mean (SD))	18.9 (4.35)	21.3 (3.66)	0.20
Bristol stool scale (median (IQR))	6.0 (4.0–6.5)	6.0 (3.0–6.0)	0.97
FCP µg/g (median (IQR))	1208.0 (1023.5–3086.5)	50.0 (14.5–900)	0.01
CRP mg/L (median (IQR))	<2.5 (<2.5–39.5)	<2.5 (<2.5–4.35)	0.15
Sample weight feces mg (median (IQR))	500.5 (485.3–512.5)	502.5 (486.3–508.8)	0.88
Sample weight urine mg (median (IQR))	644.5 (532.8–726.0)	628.5 (592.0–663.3)	0.79
Storage time months (median (IQR))	6.5 (5.8–6.5)	10 (6.75–15.5)	0.14
Physician’s Global Assessment			
Quiescent	0		
Mild	6		
Moderate	4		
Severe	0		
Crohn’s Disease (n = 5) localization			
Ileal (L1)	2		
Colonic (L2)	1		
Ileocolonic (L3)	2		
Proximal disease (L4)	1		
Crohn’s Disease behavior			
B1 (NSNP)	4		
B1p (NSNP +p)	1		
B2 (S)	0		
B2p (S+p)	0		
B3 (P)	0		
B3p (P+p)	0		
Ulcerative Colitis (n = 5) localization			
Proctitis (E1)	0		
Left sided (E2)	2		
Extensive (E3)	3		

Values were obtained at inclusion. CD and UC localization and behavior were determined using the Paris classification, based on findings during ileocolonoscopy and esophagogastroduodenoscopy and magnetic resonance enteroclysis before start of treatment [26]. Abbreviations: CRP, C-reactive protein; FCP, fecal calprotectin; IQR, interquartile range; SD, standard deviation; NSNP, non stricturing non-penetrating; S, stricturing; P, penetrating; p, perianal disease.

**Table 2:** Diagnoses controls.

Diagnosis	Number
Irritable bowel syndrome	2
Functional abdominal pain	1
Helicobacter pylori infection	1
Juvenile polyp	1
Multiple angiodysplasia	1
IBD excluded without alternative diagnosis	4

All diagnoses in the controls were established after diagnostic endoscopy. In four children, no diagnosis was established. Abbreviations: IBD, inflammatory bowel disease.

**Table 3:** Medication usage.

IBD	n	Controls	n
Number of participants receiving medication	3		6
Type of prescribed medication			
Ferrous fumarate	1	Macrogol	2
Ethinylestradiol/Desogestrel	1	Ibuprofen/Naproxen	2
Acrivastine	1	Formoterol/Beclamethason *	1
		Mebeverine	1
		Omeprazole	1
		Montelukast	1
		Ferrous fumarate	1
		Ondansetron	1
		Methylphenidate	1

All values were obtained at inclusion. In the control group, more medication usage was reported. Several participants were prescribed more than one type of medication. Abbreviations: n, number; IBD, inflammatory bowel disease; *, inhaler.

## Supplementary Materials

The following are available online at https://www.mdpi.com/1424-8220/19/20/4496/s1, **Table S1.** Statistical analyses of fecal VOC profiles for the discrimination of IBD from controls; **Table S2.** Statistical analyses of urinary VOC profiles for the discrimination of IBD from controls.
