# Supplementary material for: Simultaneous Assessment of Urinary and Fecal Volatile Organic Compound Analysis in De Novo Pediatric IBD
Source: Sensors (Basel). 2019 Oct 16;19(20):4496. doi: 10.3390/s19204496 (PMC6832645; doi:10.3390/s19204496)
Supplement: Supplementary file 1 [file sensors-19-04496-s001.pdf]

Table S1. Statistical analyses of Fecal VOC profiles for the discrimination of IBD from controls

	AUC [95% CI]	Sensitivity [95% CI]	Specificity [95% CI]	PPV	NPV	p-value	Threshold
Sparse logistic regression	0.53 [0.25-0.81]	0.4 [0.12-0.74]	0.8 [0.44-0.97]	0.67	0.57	0.602	0.140
Random forest	0.53 [0.24-0.82]	0.3 [0.067-0.65]	1.0 [0.69-1.00]	1.00	0.59	0.425	0.936
Gaussian Process	0.69 [0.44-0.94]	0.9 [0.55–1.00]	0.5 [0.19-0.81]	0.64	0.83	0.083	0.435
Support Vector Machine	0.60 [0.34-0.86]	0.8 [0.44-0.97]	0.5 [0.19-0.81]	0.62	0.71	0.218	0.618
Neural Network	0.73 [0.47-0.99]	0.7 [0.35-0.93]	0.9 [0.55-1.00]	0.88	0.75	*0.038*	0.499

Here, all the statistical analyses performed on the fecal VOC profiles are listed. For these analyses, 100 features were used in the 10 fold cross validation. The neural network analysis is identified as the best performing algorithm for the discrimination of IBD from controls based on fecal VOC profiles. **Abbreviations:** AUC, area under the curve; CI, confidence interval; PPV, positive predictive value; NPV, negative predictive value; IBD, inflammatory bowel disease; VOC, volatile organic compounds.

Table S2. Statistical analyses of urinary VOC profiles for the discrimination of IBD from controls

	AUC [95% CI]	Sensitivity [95% CI]	Specificity [95% CI]	PPV	NPV	p-value	Threshold
Sparse logistic regression	0.78 [0.57-1.00]	0.8 [0.44-0.97]	0.7 [0.35-0.93]	0.73	0.78	*0.028*	0.502
Random forest	0.69 [0.44-0.94]	1.0 [0.69–1]	0.5 [0.19-0.81]	0.67	1.00	0.070	0.876
Gaussian Process	0.35 [0.09-0.61]	0.6 [0.26-0.88]	0.5 [0.19-0.81]	0.55	0.56	0.879	0.542
Support Vector Machine	0.47 [0.19-0.75]	0.4 [0.12-0.74]	0.8 [0.44-0.97]	0.67	0.57	0.604	0.608
Neural Network	0.41 [0.14-0.68]	0.6 [0.26-0.88]	0.5 [0.19-0.81]	0.55	0.56	0.764	0.293

Here, all the statistical analyses performed on the urinary VOC profiles are listed. For these analyses, 100 features were used in the 10 fold cross validation. The Sparse logistic regression analysis is identified as the best performing algorithm for the discrimination of IBD from controls based on fecal VOC profiles. **Abbreviations:** AUC, area under the curve; CI, confidence interval; PPV, positive predictive value; NPV, negative predictive value; IBD, inflammatory bowel disease; VOC, volatile organic compounds.
